# Enhancing Inflammatory Factors, Nitric Oxide, and Arterial Stiffness Through Aquatic Walking for Amelioration and Disease Prevention: Targeting in Obese Elderly Women

**DOI:** 10.1155/mi/5520987

**Published:** 2024-12-23

**Authors:** Woo-Hyeon Son, Woo-Min Jeong, In Young Park, Min-Seong Ha

**Affiliations:** ^1^Institute of Convergence Bio-Health, Dong-A University, 26 Daesingongwon-ro, Seo-gu, Busan 49201, Republic of Korea; ^2^Department of Sport and Leisure Studies, Gimcheon University, 214 Daehak-ro, Gimcheon-si, Gyeongsangbuk-do 39528, Republic of Korea; ^3^Undergraduate Liberal Arts College, Tongmyong University, 428 Sinseon-ro, Nam-gu, Busan 48520, Republic of Korea; ^4^Laboratory of Sports Conditioning: Nutrition Biochemistry and Neuroscience, Department of Sport Science, College of Arts and Sports, University of Seoul, 163 Seoulsiripdaero, Dongdaemun-gu, Seoul 02504, Republic of Korea

**Keywords:** aquatic walking, baPWV, CVD, IL-6, NO, obese elderly women, TNF-*α*

## Abstract

In elderly women, hormonal changes lead to elevated body fat content, which results in elevated levels of vascular inflammatory factors, thereby increasing the risk of cardiovascular diseases (CVDs) associated with endothelial dysfunction. Regular physical exercises tend to keep these in check and are protective to the body. Aerobic exercise has been reported to improve CVD in obese elderly women; in this regard, aquatic exercises have been demonstrated to be more efficient in energy metabolism than land-based exercise. This study aimed to examine the effect of aquatic walking exercises on the levels of inflammatory factors, nitric oxide (NO), and brachial–ankle pulse wave velocity (baPWV) in obese elderly women. We measured these in 26 obese elderly women who were randomly assigned to control (*n* = 12) and aquatic walking exercise (*n* = 14) groups. After subjecting them to aquatic walking exercises thrice a week for 12 weeks, we specifically found a significant reduction in IL-6 levels and an increase in NO levels in these obese elderly women. This was paralleled with a reduction in the right baPWV (baPWV-R). Together, these results indicate that aquatic walking exercises can help improve vascular inflammatory factors, NO levels, and arterial stiffness.

## 1. Introduction

Elderly women experience a gradual decline in sex hormone levels [1], which leads to increased abdominal fat mass and decreased lean body mass, thereby increasing the risk of obesity [2, 3]. Obesity is an important cause of cardiovascular disease (CVD) associated with endothelial dysfunction, such as atherosclerotic disease [4]. The accumulation of adipose tissue leads to a marked elevation in the levels of pro-inflammatory factors, specifically tumor necrosis factor-alpha (TNF-*α*) and interleukin-6 (IL-6), within the body [5, 6].

Increased levels of TNF-*α* and IL-6 are identified as risk factors for the development of atherosclerosis and CVD [7]. In a cross-sectional study, Schmidt et al. [8] demonstrated that obese individuals had high levels of inflammatory cytokines. Excessive TNF-*α* secretion influences the induction of atherosclerosis via the accumulation of cholesterol in the endothelium [9] and inhibits the production of nitric oxide (NO), a vasodilator, in vascular endothelial cells, thereby also triggering endothelial dysfunction [10, 11].

TNF-*α* stimulates the secretion of IL-6 [12], an independent predictor of CVD; increased IL-6 levels in the body are closely associated with CVD-related mortality and morbidity [13]. Hung et al. [14] reported that IL-6 directly affected endothelial cells by lowering the activation of endothelial NO synthase (eNOS), an enzyme responsible for NO production. Therefore, an increase in the levels of inflammatory cytokines due to obesity decreases NO production and bioavailability [15], and the resulting endothelial dysfunction decreases arterial compliance, increases central pulse pressure to facilitate arterial stiffness, and increases the risk of CVD [16, 17].

Regular physical exercises have an anti-inflammatory effect by suppressing the onset of inflammation in blood vessels and reducing the risk of CVD [18]. Aerobic exercise has proven to be a suitable intervention to improve the levels of inflammatory cytokines in obese elderly women [19]. Aquatic walking may be particularly beneficial for individuals with obesity and the elderly, owing to its low impact on knee and ankle joints. This reduced impact is attributed to the buoyancy experienced during underwater movements, which potentially mitigates the risk of acute joint injuries [20]. Furthermore, water resistance enhances energy metabolism efficiency during limb movement [21]. Kurobe et al. [22] reported higher energy consumption in aquatic walking compared to land-based exercise (walking or cycling). Several studies suggest that aquatic exercise can improve inflammatory markers and cardiovascular health in elderly individuals [23, 24]. Our previous study demonstrated that habitual aquatic walking positively affects arterial stiffness in the elderly population [25]. Yet, the effects of aquatic walking exercise on vascular inflammation and NO remains largely unexplored. We hypothesized that aquatic walking exercises offer therapeutic benefits by ameliorating inflammatory markers, enhancing NO production, and reducing arterial stiffness in obese elderly women. To validate this proposition, we conducted a detailed 12-week study probing the influence of such exercises on vascular inflammation, dynamics of NO metabolism, and the elasticity of the arteries.

## 2. Methods

### 2.1. Participants

**Figure 1:**
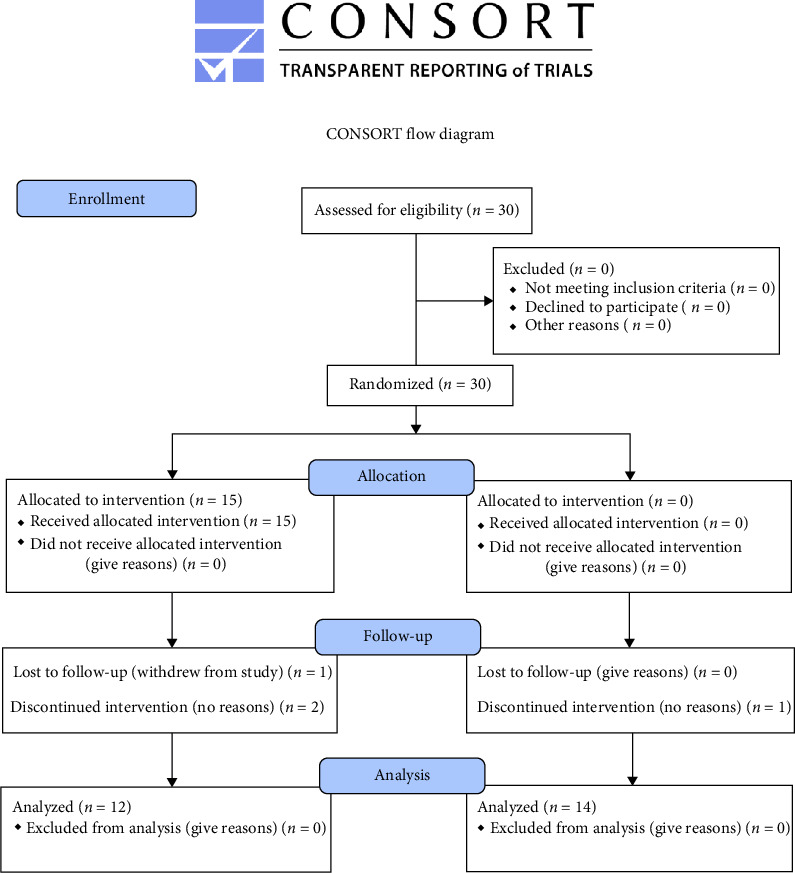
Study design and CONSORT flow diagram for the individual randomized controlled trial. Twenty-six participants, excluding dropouts, were randomly assigned to the control (*n* = 12) and exercise (*n* = 14) groups. The aquatic walking program was conducted thrice a week for 12 weeks, and exercise intensity was performed at RPE 11–12 in weeks 1–6 and RPE 13–14 in weeks 7–12. The following outcomes were measured at baseline (pre) and after 12 weeks (post) of sessions: body composition, IL-6, TNF-*α*, NO, and arterial stiffness. IL-6, interleukin-6; NO, nitric oxide; RPE, rating of perceived exertion; TNF-*α*, tumor necrosis factor-alpha.

Obese elderly women (body mass index [BMI] ≥25 kg/m^2^, no menstrual periods ≥12 months) were included for this study. They had not performed regular physical activity for 6 months before the study and did not have skeletal diseases. They were informed about the study's purpose and potential benefits and risks, and informed consent was obtained from each participant. All participants were divided into two groups—control (CON, *n* = 15) and aquatic exercise (EX, *n* = 15). Three and one patient dropped out in the CON and EX groups, respectively (Figure 1). The sample size of our study participants was calculated using G-power 3.1 (University of Kiel, Kiel, Germany). The estimated sample size was determined through an *F*-test ANOVA for repeated measures with (effect size: 0.25 [default], significance level: 0.05, power: 0.65), resulting in a total of 26 participants. Taking dropout rates into account, we recruited a total of 30 subjects. Participants were recruited and randomized to two groups. The characteristics of the participants are presented in Table 1. This study was approved by the Institutional Review Board designated by the Ministry of Health and Welfare in South Korea (2-1040709-AB-N-01-202109-HR-066-04), and the study was conducted in accordance with the Helsinki Declarations and retrospectively registered with the Clinical Research Information Service (CRIS) in the Republic of Korea (KCT0008621, July 17, 2023, https://cris.nih.go.kr).

### 2.2. Study Design

Individuals in the EX group participated in the aquatic walking exercise program for 12 weeks; the program consisted of a warm-up, main exercise, and cool-down. The CON group did not take part in this program and performed no such regular physical activity (≤30 min/day, ≤2 days/week).

### 2.3. Aquatic Walking Program

Aquatic walking was performed in a 25-m pool with a 1.2-m water depth and 28–29°C water temperature thrice a week (Monday, Wednesday, and Friday) for 12 weeks. The program consisted of a 5-min warm-up, 40-min main exercise, and 5-min cool-down, amounting to 50 min overall. The warm-up involved forward and backward walking, tip-toe walking, and stretching. The main exercise was aquatic walking. The cool-down involved stretching. The exercise intensity was based on the Borg rating of perceived exertion (RPE) [26] RPE 11–12 for weeks 1–6 and RPE 13–14 for weeks 7–12.

### 2.4. Blood Sample Analysis

Blood collection and other measurements were performed on each participant at the same time in the morning (7:00–9:00 AM), after fasting from 10 PM the previous evening, before and after the 12-week intervention. Using an EDTA tube and a needle, 5 mL of blood was collected from the antebrachial vein, centrifuged for 10 min at 3000 rpm, and the isolated plasma was stored at ≥−70°C for subsequent analysis.

To quantify IL-6 and TNF-*α* levels, we performed an enzyme-linked immunosorbent assay using the Quantikine HS Human IL-6 and Quantikine HS Human TNF-*α*, respectively (R&D, Minneapolis, USA). The absorbance at 450 nm was analyzed using the Microplate Reader VERSA Max (Molecular device, California, USA) and calculated, as previously described [27].

To quantify NO production, we used the Griess Reagent method with a Nitrate/Nitrite Colorimetric Assay Kit (Cayman Chemical, Michigan, USA) and GENios (TECAN, Männedorf, Switzerland) for 540 nm absorbance, calculated as previously described [28].

### 2.5. Arterial Stiffness

For measurement of the brachial–ankle pulse wave velocity (baPWV), we used the VP-1000 plus (Colin Co. Ltd, Komaki, Japan). Electrodes were attached to the left substernal region and the inner area of the left and right wrists of the participant after supine for 10 min rest. Cuffs were wrapped around the upper arms and the ankles on both sides [29].

### 2.6. Anthropometrics

Participants were instructed to stand barefoot to ensure accuracy and consistency in the measurements. Height measurement was meticulously conducted using a state-of-the-art automatic measuring device (InLabS50, Inbody, Seoul, Korea).

### 2.7. Statistical Analysis

SPSS Statistics 27.0 (IBM Corp, Armonk, New York, USA) and GraphPad Prism (GraphPad Software, Inc., San Diego, CA) were used for statistical analysis. Data normality was checked using the Shapiro–Wilk test, and the effects of aquatic walking exercise on vascular inflammatory factors, NO, and arterial stiffness were identified using two-way repeated measures ANOVA with treatment (EX and CON) and time (pre- and post-) as independent variables. The Bonferroni test was used for post hoc analysis. All data were expressed using means and standard deviations, and the significance level was set at *p* < 0.05.

## 3. Results

### 3.1. Effects of the 12-Week Aquatic Walking Exercise on Vascular Inflammatory Factors and NO

**Figure 2:**
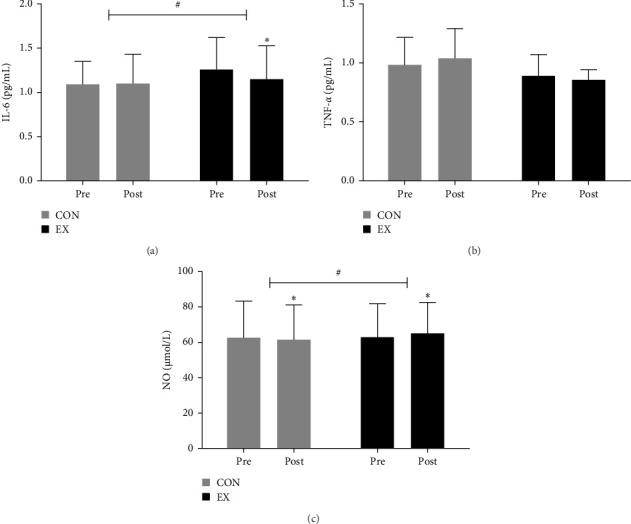
Effect of aquatic walking on blood results in obese elderly women (a) IL-6 decreased significantly in the EX group compared to the CON group (time effect: *p* < 0.05, interaction effect: *p* < 0.05). (b) TNF-*α* did not differ significantly between the EX and CON groups. (c) NO increased significantly in the EX group compared to the CON group (time effect: *p* < 0.05, interaction effect: *p* < 0.05). *⁣*^*∗*^Indicates significant differences between preintervention and post-intervention results (*p* < 0.05), ^#^Indicates significant interaction effects (*p* < 0.05). CON, control group; EX, exercise group; IL-6, interleukin-6; NO, nitric oxide; TNF-α, tumor necrosis factor-alpha.

As illustrated in Figure 2 and Supporting Information: Table S1, the 12-week aquatic walking intervention yielded notable changes in vascular inflammatory factors and NO concentrations among obese elderly women.

#### 3.1.1. IL-6

There was a significant reduction in IL-6 levels in the EX group from 1.26 ± 0.33 to 1.15 ± 0.35 pg/mL (*p* < 0.05). This change manifested with a noteworthy interaction effect (*F* = 4.354; *p* < 0.05).

#### 3.1.2. TNF-α

While the CON group exhibited an increase in TNF-*α* levels from 0.98 ± 0.24 to 1.04 ± 0.25 pg/mL, the EX group exhibited a decline from 0.89 ± 0.16 to 0.85 ± 0.08 pg/mL. However, these fluctuations were not statistically significant.

#### 3.1.3. NO

The CON group exhibited a decrease in NO levels from 62.51 ± 20.81 to 61.63 ± 19.60 μmol/L. However, the EX group exhibited a statistically significant increase in NO level from 62.87 ± 17.47 to 64.86 ± 16.45 μmol/L (*p* < 0.05), with a significant interaction effect (*F* = 4.520; *p* < 0.05).

### 3.2. Impact of the 12-Week Aquatic Exercise on Arterial Stiffness

**Figure 3:**
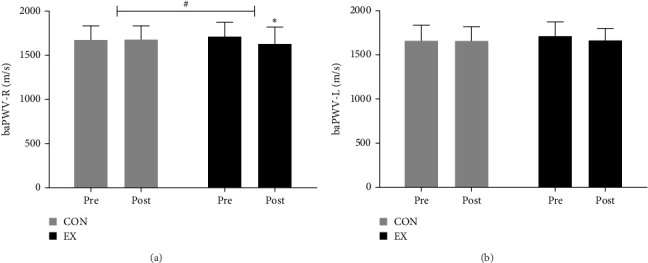
Effect of aquatic walking on arterial stiffness in obese elderly women (a) baPWV-R decreased significantly in the EX group compared to the CON group (time effect: *p* < 0.05, interaction effect: *p* < 0.05). (b) baPWV-L was not significantly different in the EX group compared to the CON group. *⁣*^*∗*^Indicates significant differences between preintervention and postintervention results (*p* < 0.05), ^#^Indicates significant interaction effects (*p* < 0.05). baPWV-L, brachial–ankle pulse wave velocity (left); baPWV-R, brachial–ankle pulse wave velocity (right); CON, control group; EX, exercise group.

Figure 3 and Supporting Information: Table S2 details the implications of the aquatic exercise regimen on arterial stiffness in our study population.

#### 3.2.1. baPWV (Right) (baPWV-R)

The CON group exhibited a slight increase in baPWV-R from 1671.08 ± 155.34 to 1675.17 ± 154.58 m/s, which was not statistically significant; however, the EX group exhibited a significant decrease from 1708.29 ± 153.11 to 1625.00 ± 176.15 m/s (*p* < 0.05), with a significant interaction effect (*F* = 4.791;*p* < 0.05).

#### 3.2.2. baPWV (Left) (baPWV-L)

The CON group exhibited an increase in baPWV-L from 1654.83 ± 179.60 to 1657.08 ± 158.86 m/s; the EX group exhibited a decrease from 1728.29 ± 147.78 to 1659.07 ± 131.86 m/s. However, these changes lacked statistical significance.

## 4. Discussion

We assessed the potential benefits of a 12-week aquatic walking regimen on inflammatory markers, NO levels, and arterial stiffness in obese elderly women. We hypothesized that the aquatic exercise intervention would manifest improvements in IL-6, TNF-*α*, and NO dynamics, notably attenuating arterial stiffness. The empirical findings reveal a significant reduction in IL-6 levels and baPWV, complemented by a significant amplification in NO levels, thereby affirming our initial hypothesis.

The reduced estrogen levels in elderly women lead to increased fat mass and decreased lean body mass, contributing to weight gain and obesity [30]. An augmentation in obesity and adipose tissue mass correlates with elevated concentrations of pro-inflammatory cytokines, including TNF-*α* and IL-6 [6]. Park, Park, and Yu [31] demonstrated a strong correlation between body fat mass and inflammatory cytokines. An increase in inflammatory cytokines causes atherosclerosis, arterial stiffness, and endothelial dysfunction [16, 32] and increases the risk of CVD [33]. TNF-*α* is a protein mediator of the inflammatory response in the early stages, with a role in human defense mechanism that includes inflammatory reactions and tissue recovery and regeneration [34, 35]. However, TNF-*α* overproduction and secretion due to increased body fat contribute to vascular inflammation [36, 37] and endothelial dysfunction [38].

Regular exercise is shown to effectively modulate the levels of TNF-*α*, a recognized risk factor for CVD [39]. A previous study reported that walking exercises reduce TNF-*α* levels in obese participants [40], and 3-week water-walking and water-bike endurance exercises decrease TNF-*α* levels in patients with multiple sclerosis [41]. These exercises training could potentially contribute to reducing excessive body fat, a source of inflammatory cytokines, via increased energy expenditure resulting from heightened water resistance [42]. In contrast, Beavers et al. [43] reported no change in TNF-*α* in older adults after a 12-month physical activity intervention. Despite no significant between-group variations in the present study, TNF-*α* was slightly lower in the EX group, suggesting a potential effect of regular aquatic walking exercises in improving TNF-*α*. In this study, despite the lack of significant differences between groups, TNF-*α* levels were slightly lower in the EX group. This suggests that aquatic walking may be related to a reduction in cytokine expression, even without systemic changes in TNF-*α* levels [44]. Therefore, this suggests that regular aquatic walking exercise may have a potentially beneficial effect on improving TNF-*α* levels.

IL-6 is a cytokine secreted by adipose tissues, playing a pivotal role in the regulation of inflammatory responses [45]. It is consistently observed at elevated levels in states of obesity [45]. A prior study documented a correlation between BMI and plasma IL-6 levels in obese elderly women [46]. An increase in IL-6 is associated with increased CVD mortality and morbidity [47]; mortality was reported to be higher with plasma IL-6 ≥5 ng/mL than with IL-6 < 5 ng/mL [48]. However, IL-6 levels decrease with regular exercise [49]. Tudor-Locke and Bassett [50] and Bassett suggested that above-moderate walking (e.g., about 100 steps per minute) exercise is necessary to reduce IL-6 levels.

Ortega et al. [51] reported that an 8-month aquatic exercise intervention reduced the IL-6 levels in patients with fibromyalgia. The hypothesized mechanism driving the anti-inflammatory benefits of aquatic exercise is threefold. The inherent resistance offered by water augments exercise performance and serves as a foundational component of these benefits [52]. Concurrently, the amplified water pressure and the systematic weight reduction facilitated by regular exercise are believed to synergistically reinforce this mechanism [53]. Furthermore, it is proposed that the effects of aquatic walking exercise are mediated by a reduction in angiotensin-II, a key regulator involved in vasoconstriction, blood pressure elevation, and the expression of IL-6 [54, 55].

In the current study, a notable reduction in IL-6 levels was observed in the EX group. This finding indicates that regular aquatic walking, which facilitates weight loss, may play a role in modulating IL-6 levels. Consequently, aquatic walking could be effective in ameliorating inflammatory markers and potentially aiding in the prevention of atherosclerosis among obese elderly women.

The increase in inflammatory cytokines due to obesity promotes atherosclerosis and induces arterial stiffness and endothelial dysfunction, thereby increasing the risk of CVD [16, 56]. A previous study reported that inflammatory cytokines are associated with reduced NO bioavailability [57, 58]. NO is a vasodilator released during the conversion of L-arginine to citrulline with the endothelial isoform of eNOS in the vascular endothelium [59]. Elevated levels of inflammatory cytokines reduce NO production [60] and eNOS expression [61] in endothelial cells, and an obese state is reported to reduce NO bioavailability in animal models and humans [62]. NO reduction decreases blood flow and increases vasoconstriction [63] and blood pressure [64]. Exercise promotes eNOS expression and increases NO production and bioavailability [65]. Khalid et al. [66] reported that an 8-week walking exercise intervention increased NO levels in menopausal women, and so did a 12-week aquatic treadmill training in sedentary adults [67].

The mechanisms of NO improvement through aquatic exercise involve increasing blood flow and shear stress due to exercise, leading to the stimulation of eNOS via calcium (Ca^2+^) flowing in through the Ca^2+^ channels, with increased NO release from endothelial cells [68, 69]. This is suggested to be due to the increased venous return caused by the rise in hydrostatic in the water [21]. The NO level increased significantly in the EX group in the present study, suggesting that regular aquatic walking improves endothelial functioning, blood flow, and vascular function.

Obesity is associated with impaired vascular function and structure, endothelial dysfunction, and increased arterial medial wall thickness and arterial stiffness [70]. The increased inflammatory cytokines and decreased NO caused by obesity negatively impact vascular structure due to increased arterial wall thickness and decreased elasticity [71]. In contrast, a previous study reported that an increase in NO levels was reported as an independent factor for reducing baPWV [72]. baPWV is a well-known, noninvasive method to assess the level of vascular damage in arterial stiffness [73]. Increased baPWV is associated with endothelial dysfunction [74]. Regular aerobic exercise effectively improves PWV [75]. Our previous study demonstrated a decrease in PWV after habitual aquatic walking [25]. Park, Kwak, and Pekas [76] suggested that regular aquatic walking could reduce PWV in patients with peripheral artery disease. It is suggested that this is a synergistic effect of improvements in the sympathetic nervous system and endothelial function after underwater exercise [77, 78], combined with the enhancement of endothelial function and microvascular function following exercise in warm water (28–30°C) [76].

Furthermore, it has been proposed that the increase in NO induced by aquatic walking exercise may contribute to the reduction in the synthesis and secretion of matrix metalloproteinase-9 (MMP-9), which is recognized as an independent predictor of aortic stiffness [79, 80].

In the current study, a significant reduction in baPWV-R was noted in the EX group. Although no statistically significant decrease was observed in the baPWV-L within the EX group, a trend toward reduction was evident. It is suggested that regular aquatic walking exercise could improve IL-6 and increase NO production and bioavailability to reduce arterial stiffness, potentially contributing to the amelioration and prevention of CVD in obese elderly women.

The results of this study should be interpreted with caution due to several limitations that may affect their generalizability. First, the number of participants was relatively small. Second, the currently administered drugs, diet, and physical activity could not be controlled. Third, the results may not apply to the general population as the participants were obese elderly women. Based on our research, there is a clear need for further studies that incorporate larger sample sizes and diverse age groups for a more comprehensive analysis. Fourth, flow-mediated dilation was not investigated for the evaluation of endothelial function.

This study has several methodological limitations. First. We were unable to regulate the daily activities of the participants. This may potentially impact the efficacy of the aquatic walking exercise program. Second, we did not monitor dietary intake. Further research is needed because this could play a significant role in improving inflammatory markers. Last, the sample size is limited to 26 participants. This may not be generalized to the entire population, and additional studies with more participants are necessary.

## 5. Conclusions

Aquatic walking is a safe and useful therapeutic intervention to improve IL-6, NO, and baPWV levels in obese elderly women. Using these mechanisms, regular aquatic walking could be expected to improve vascular inflammatory factors and vasodilator levels and could be in the amelioration and/or prevention of CVD, including atherosclerosis, in obese elderly women.

## Figures and Tables

**Table 1:** Characteristics of participants.

Variables	CON (*n* = 12)		EX (*n* = 14)	
	Pre	Post	Pre	Post
Age (years)	71.17 ± 4.66	—	71.67 ± 2.74	—
Height (cm)	153.82 ± 4.66	—	153.58 ± 4.93	—
Weight (kg)	63.87 ± 4.89	64.18 ± 4.92*⁣*^*∗∗*^	63.33 ± 4.98	62.71 ± 5.31
BMI (kg/m^2^)	27.30 ± 2.63	27.14 ± 1.91	26.86 ± 1.89	26.59 ± 1.96
Underlying diseases				
Hypertension	5		4	
Dyslipidemia	3		5	
Diabetes mellitus	4		3	
Osteoporosis	4		3	
Medications				
ACE inhibitors	5		4	
Statin	3		5	
Diabetic medication/insulin therapy	4		3	
Bisphosphonate	4		3	

*Note:* Values are presented as mean (M) ± standard deviation (SD).

Abbreviations: ACE, angiotensin-converting enzyme; CON, control group; EX, exercise group.

*⁣* ^*∗∗*^Indicates significant differences between pre-intervention and post-intervention results (*p* < 0.01).

## Data Availability

The authors declare that all data and materials are available for sharing upon formal request to the corresponding author.

## Supporting Information

Supporting Information Table S1: Effect of aquatic walking on inflammatory factors in obese postmenopausal women, Table S2: Effect of aquatic walking on arterial stiffness in obese postmenopausal women.
