# Supplementary material for: Enhancing Inflammatory Factors, Nitric Oxide, and Arterial Stiffness Through Aquatic Walking for Amelioration and Disease Prevention: Targeting in Obese Elderly Women
Source: Mediators Inflamm. 2024 Dec 23;2024:5520987. doi: 10.1155/mi/5520987 (PMC11685319; doi:10.1155/mi/5520987)
Supplement: Supporting Information — Table S1: Effect of aquatic walking on inflammatory factors in obese postmenopausal women, Table S2: Effect of aquatic walking on arterial stiffness in obese postmenopausal women. [file 5520987.f1.pdf]

Table S1. Effect of aquatic walking on inflammatory factors in obese postmenopausal women

Variables	CON (*n* = 12)		EX (*n* = 14)		Source	F-value (*P*-value)
	M	SD	M	SD		
					time	2.119 (0.160)
	1.09	0.26	1.10	0.33		
IL-6 (pg/mL)					group	0.714 (0.407)
	1.26	0.36	1.15	0.38*		
					time*group	4.354 (0.049)
					time	0.030 (0.864)
	0.98	0.24	1.04	0.25		
TNF-α (pg/mL)					group	3.199 (0.087)
	0.89	0.18	0.85	0.09		
					time*group	3.774 (0.065)
					time	0.667 (0.423)
	62.51	20.81	61.63	19.60*		
NO (μmol/L)					group	0.052 (0.822)
	62.87	18.99	64.86	17.91*		
					time*group	4.520 (0.045)

Values are presented as mean ± standard deviation. *P<.05 vs. pre
IL-6: interleukin-6; TNF- α: tumor necrosis factor-alpha; NO: nitric oxide; CON: control group; EX: exercise group; SD: standard deviation

Table S2. Effect of aquatic walking on arterial stiffness in obese postmenopausal women

Variables	CON (*n* = 12)		EX (*n* = 14)		Source	F-value (*P*-value)
	M	SD	M	SD		
					time	3.937 (0.060)
	1671.08	155.34	1675.17	154.58		
baPWV(R) (m/s)					group	0.010 (0.922)
	1708.25	166.45	1625.00	191.49*		
					time*group	4.791 (0.040)
					time	2.630 (0.119)
	1654.83	179.60	1657.08	158.86		
baPWV(L) (m/s)					group	0.362 (0.554)
	1708.25	160.59	1659.08	143.29		
					time*group	2.997 (0.097)

Values are presented as mean (M) ± standard deviation (SD). *P<.05 vs. pre
baPWV(R): brachial-ankle pulse wave velocity (right); baPWV(L): brachial-ankle pulse wave velocity (left); CON: control group; EX: exercise group; SD: standard deviation
